# Protocol for the visualization of pRps6-positive cells in larval zebrafish brains using whole-mount immunofluorescence and light-sheet microscopy

**DOI:** 10.1016/j.xpro.2024.103587

**Published:** 2025-01-18

**Authors:** Olga Doszyn, Tomasz Dulski, Justyna Zmorzynska

**Affiliations:** 1 Laboratory of Developmental Neurobiology, International Institute of Molecular Mechanisms and Machines, 02-247 Warsaw, Poland; 2 Laboratory of Molecular and Cellular Neurobiology, International Institute of Molecular and Cell Biology in Warsaw, 02-109 Warsaw, Poland

**Keywords:** developmental biology, microscopy, model organisms, neuroscience

## Abstract

Due to their small size and transparency, larval zebrafish are a useful model for whole-brain imaging. Here, we present a protocol for the visualization of phosphorylated Rps6, a marker of mechanistic target of rapamycin complex 1 (mTORC1) activity, in the zebrafish brains at 5 days post fertilization (dpf), using whole-mount immunofluorescence and light-sheet microscopy. We describe steps for sample preparation, storage, staining, and imaging. This protocol can also be modified for staining with antibodies against other proteins.

For complete details on the use and execution of this protocol, please refer to Doszyn et al.^1^

## Highlights

• Instructions for whole-mount immunofluorescence staining in larval zebrafish brains
• A step-by-step guide for the visualization of phosphorylated Rps6 protein in the brain
• Procedures for the evaluation of mTORC1 activity in larval zebrafish brains
• Guidelines for larval zebrafish brain imaging with light-sheet microscopy

## Abstract (editor highlights)

Publisher’s note: Undertaking any experimental protocol requires adherence to local institutional guidelines for laboratory safety and ethics.

## Graphical abstract

**Figure:**
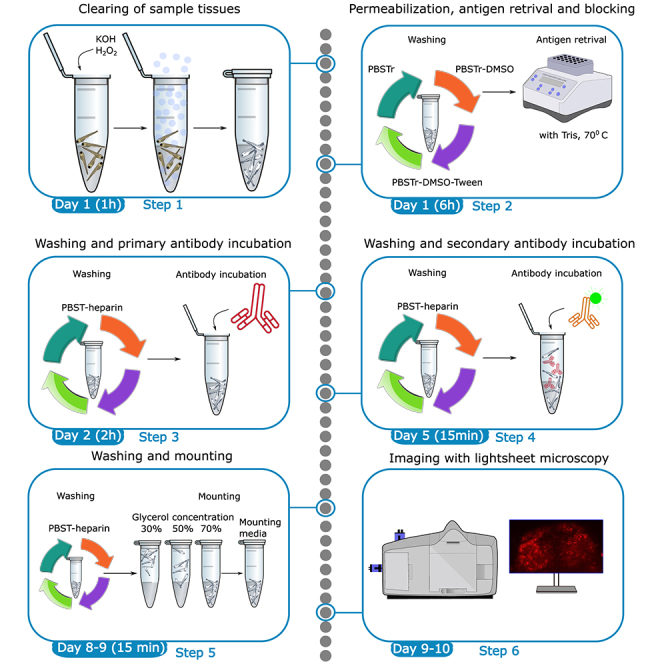

## Before you begin

In this protocol, we provide detailed instructions for whole-mount immunofluorescence staining in larval zebrafish brains. The example provided here is visualization of phosphorylated Rps6 (Ribosomal S6 Protein) in the brain. As Rps6 is a downstream target of mTORC1 (Mechanistic Target of Rapamycin Complex 1), it can be used as an indicator of mTORC1 activity. However, we have used this protocol for all of our immunofluorescence staining experiments, and it can be adapted for staining against other proteins. Prior to the staining procedure, zebrafish larvae were bred and maintained according to international standards, and genotyped using methods described by Kedra et al.^2^ and Doszyn et al.^3^ We have used this protocol on zebrafish larvae up to 14 dpf; for older fish, additional optimalization (such as extending incubation times necessary for tissue penetration) might be required.

### Institutional permissions

For the procedure described here, the *tsc2*^*vu242/+*^ zebrafish line was used. All experiments performed were conducted in accordance with the Act of 15 January 2015 on the protection of animals used for scientific and educational purposes, Directive 2010/63/EU of the European Parliament and of the Council of 22 September 2010 on the protection of animals used for scientific purposes and were approved by the Animal Welfare Commission of the IMol and the IIMCB.

Any experiments using zebrafish older than 5 dpf require the permission of local ethical committee.

### Preparation of buffers and stock solutions

**Timing: 1–2 h**

1. Prepare 20× Tricaine stock solution for larvae euthanasia.
  a. Dissolve 1 g of Tricaine in 244.75 mL of MilliQ H_2_O.
  b. Dissolve 60.57 g of Tris base in 450 mL of MilliQ H_2_O to obtain a 1 M solution. Adjust the pH to 9 with HCl, and top up the volume to 500 mL with MilliQ H_2_O.
  c. Add 5.25 mL of 1 M Tris buffer to the Tricaine solution.
  d. Store at 4°C.
2. Prepare TE buffer for DNA extraction (if genotyping of larvae is required).
  a. Dissolve 60.57 g of Tris base in 450 mL of MilliQ H_2_O to obtain a 1 M solution. Adjust the pH to 8 with HCl, and top up the volume to 500 mL with MilliQ H_2_O.
  b. Dissolve 73.06 g of EDTA in 450 mL of MilliQ H_2_O to obtain a 0.5 M solution. Adjust the pH to 8 with NaOH, and top up the volume to 500 mL with MilliQ H_2_O.
  c. Add 5 mL of the Tris solution and 1 mL of the EDTA solution to 500 mL of MilliQ H_2_O.
  d. Autoclave and store at 20°C–22°C.
3. Prepare 60× stock solution of the E3 medium for the maintenance of larvae (recipe included in materials and equipment). Autoclave and keep at 4°C.
  a. Prepare the E3 medium by diluting the stock to 1× in MilliQ H_2_O.
4. Prepare 10% KOH solution for tissue clearing.
  a. Dissolve 5 g of solid KOH in 50 mL of ddH2O.
  b. Store at 4°C.
5. Prepare PBSTr solution for washing and permeabilization.
  a. Add 0.2% of Triton X-100 to PBS (e.g., add 100 μL Triton X-100 to prepare 50 mL of PBSTr solution).
  b. Store at 4°C.

**CRITICAL:** undiluted Triton X-100 should be handled under a chemical hood.

6. Prepare PBSTr-DMSO solution for washing and permeabilization.
  a. Add 0.2% of Triton X-100 and 20% DMSO to PBS (e.g., add 100 μL Triton X-100 and 10 mL DMSO to prepare 50 mL of PBSTr-DMSO solution).
  b. Store at 4°C.
7. Prepare PBSTr-DMSO-Tween solution for washing and permeabilization.
  a. Add 0.2% of Triton X-100, 20% DMSO and 0.1% TWEEN 20 to PBS (e.g., add 100 μL Triton X-100, 10 mL DMSO, and 50 μL TWEEN 20 to prepare 50 mL of PBSTr-DMSO-Tween solution).
  b. Store at 4°C.
8. Prepare 150 mM Tris buffer for antigen retrieval.
  a. Dissolve 9.0855 g of Tris base in 450 mL of ddH2O.
  b. Adjust the pH to 9 with HCl.
  c. Transfer the buffer to a volumetric flask and adjust to 500 mL with ddH2O.
  d. Transfer to a clean and sterile glass bottle.
9. Prepare 2× GDB-Tr blocking solution.
  a. Add 0.4% gelatin and 0.2% Triton X-100 to PBS (e.g., add 10 mL Gelatin solution and 100 μL Triton X-100 to prepare 50 mL of 2× GDB-Tr blocking solution).
  b. Aliquot and store at −20°C.
10. Prepare PBST-heparin solution for washing and antibody dilution.
  a. Add 0.2% TWEEN 20 and 10 μg/mL heparin salt to PBS (e.g., add 100 μL TWEEN 20 and 50 μL of prepared 10 mg/mL heparin salt stock).
  b. Store at 4°C.
11. Prepare mounting medium.
  a. Dissolve 1 g of propyl gallate in 45 mL of glycerol.
  b. Add 5 mL of PBS, mix using a magnetic stirrer.
  c. Aliquot and store at −20°C protected from light.
12. Prepare a 2% low melting point agarose solution for imaging.
  a. Dissolve low melting point agarose in 1× E3 medium to the final concentration of 2%.
  b. If not prepared immediately before imaging, aliquot and store at 4°C.
13. Prepare 36% paraformaldehyde stock for fixation.
  a. Dissolve paraformaldehyde powder in PBS to the final concentration of 36%.
  b. Aliquot and store at −20°C.

**CRITICAL:** Paraformaldehyde must be handled under a chemical hood.

### Zebrafish breeding

**Timing: 1–2 h per day for 4 days**

14. Prepare a spawning tank with male and female fish in a light-controlled room where the light is switched off overnight and switched on in the morning.
  a. If a light-controlled environment is not available, separate the fish by a divider, and remove it the following morning.
15. On the following morning, collect the eggs. Remove unfertilized eggs and debris. Place up to 60 eggs per a Ø100 mm Petri dish filled with 40 mL of 1x E3 medium. Keep at 28.5°C in an incubator set to a diurnal cycle of 14 h light/10 h darkness.
16. Every day until collection, remove dead larvae and debris, and replace the medium with fresh 1x E3 solution.

### Sample fixation

**Timing: 24 h**

**CRITICAL:** This step utilizes paraformaldehyde, which is harmful; therefore, all following steps must be performed under a chemical hood.

17. Freshly before use, prepare a 4% fixing solution of formaldehyde by diluting a 36% stock in PBS.
  a. Prepare 50 μL of fixing solution per larva. Leftover 4% solution can be stored at 4°C and used within 48 h, after which it should be discarded.

***Optional:*** If the samples will be stained against phosphorylated antibodies, add sodium fluoride (NaF) to the final concentration of 20 mM as a phosphatase inhibitor.

18. If genotyping is required, prepare two 96-well plates – one filled with 50 μL of fixing solution per well and the second – with 20 μL of TE buffer respectively.
19. Collect larvae into the fixing solution.
  a. Euthanize the larvae by adding the 20x Tricaine solution to the medium to the final concentration of 3x. If genotyping is not required, collect the larvae directly into 2 mL round or flat bottom tubes, remove remaining liquid, and add 1 mL of fixing solution.
  b. If genotyping is required, cut the larvae below the head. Collect the corresponding heads and tails into separate 96-well plates, placing the heads in the fixing solution, and the tails in TE buffer for DNA extraction and genotyping.
  c. Incubate the larvae in fixing solution overnight at 4°C for fixing.
20. Wash 3x with PBS. Store at 4°C in fresh PBS or proceed immediately to staining.

***Note:*** It is recommended to stain fixed samples within 2–3 weeks after fixing. During the storage, make sure to replenish evaporated PBS solution.

## Key resources table

**Table:** 

REAGENT or RESOURCE	SOURCE	IDENTIFIER
**Antibodies**		

Rabbit polyclonal anti-pRps6 (Ser235/236)	Cell Signaling Technology	Cat# CS4858; RRID:AB_916156
Goat anti-rabbit IgG (H + L) cross-adsorbed secondary antibody, Alexa Fluor 488	Thermo Fisher Scientific	Cat# A-11008; RRID:AB_143165

**Chemicals, peptides, and recombinant proteins**		

Tricaine	Sigma-Aldrich/Merck	Cat# E10521
PBS tablets	Carl Roth	Cat# 0890.2
NaCl (sodium chloride)	Chempur	Cat# 117941206
KCl (potassium chloride)	Chempur	Cat# 117397402
CaCl_2_ (calcium chloride)	Chempur	Cat# 118748703
MgCl_2_ × 6H_2_O (magnesium chloride hexahydrate)	Chempur	Cat# 116120500
Paraformaldehyde	Sigma-Aldrich/Merck	Cat# 158127
Sodium fluoride	Sigma-Aldrich/Merck	Cat# 221368
Potassium hydroxide, 90%	Sigma-Aldrich/Merck	Cat# 484016
Hydrogen peroxide, 30%	Carl Roth	Cat# 9681.1
Triton X-100	Sigma-Aldrich/Merck	Cat# X100
Dimethyl sulfoxide	Sigma-Aldrich/Merck	Cat# D8418
TWEEN 20	Sigma-Aldrich/Merck	Cat# P1379
Tris base	Carl Roth	Cat# 4855.3
HCl	Chempur	Cat# 115752837
EDTA	Sigma-Aldrich/Merck	Cat# EDS
NaOH	Sigma-Aldrich/Merck	Cat# S5881
Gelatin solution, type B, 2% in H_2_O	Sigma-Aldrich/Merck	Cat# G1393
Heparin sodium salt	Sigma-Aldrich/Merck	Cat# H3149
Propyl gallate	Sigma-Aldrich/Merck	Cat# P3130
Glycerol	Sigma-Aldrich/Merck	Cat# G5516
1-phenyl-2-thiourea (PTU)	Sigma-Aldrich/Merck	Cat# P7629
TopVision low melting point agarose	Thermo Fisher Scientific	Cat# R0801

**Experimental models: Organisms/strains**		

Zebrafish: tsc2^vu242/+^ (mixed strain) larvae until 5 dpf	Kim et al.^4^	RRID:ZFIN_ZDB-GENO-180906-3

**Software and algorithms**		

Fiji	Schindelin et al.^5^	https://fiji.sc/
R 4.3.2	R Foundation for Statistical Computing	https://www.Rproject.org
RStudio	Posit Software, PBC	https://posit.co/do wnload/rstudiodesktop/
ZEN2014 SP1 (black edition)	Zeiss	https://www.microshop.zeiss.com/en/u s/softwarefinder/sof tware-categories/zen-black

**Other**		

ThermoMixer C	Eppendorf	Cat# 2231001005
Lightsheet Z.1 microscope	Zeiss	https://www.zeiss.c om/microscopy/en/ products/lightmicroscopes/lightsheet-microscopes.html

## Materials and equipment

**Table:** 60x E3 medium stock solution

Reagent	Final concentration	Amount
NaCl	297 mM	17,4 g
KCl	10,7 mM	0,8 g
CaCl_2_	19,6 mM	2,18 g
MgCl_2_ × 6H_2_O	24 mM	4,89 g
MilliQ H_2_O	N/A	Fill up to 1 L
Total	N/A	1 L

Can be stored long term at 4°C.

## Step-by-step method details

***Note:*** For all steps, use 0.5–1 mL of solution per tube. When removing solutions, we recommend gently aspirating the liquid with glass Pasteur pipettes to avoid damage to the tissues, or sticking of tissue to the walls of a plastic pipette tip.

### Clearing of sample tissues

**Timing: 0.5–1 h**

**Figure 1:**
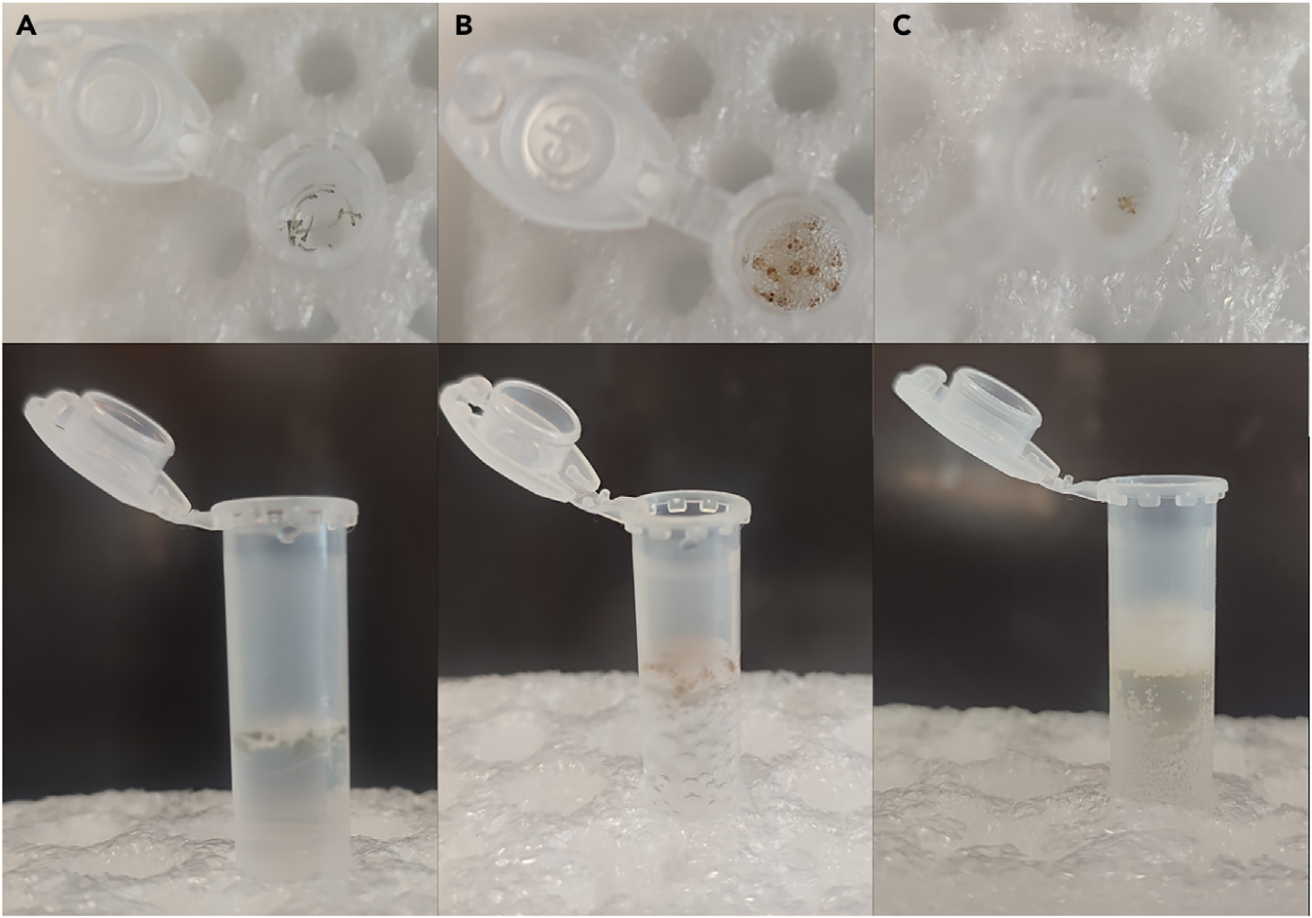
Clearing of sample tissues (A) Before adding KOH and H_2_O_2_. (B) Samples after 15 min. (C) Washed samples after 45 min. KOH removes pigmentation from the skin and eyes, in order to provide clear microscopic images. H_2_O_2_ quenches autofluorescence, reducing background noise and false positive signals during imaging.

The first step (Figure 1) removes pigmentation from the skin and eyes, in order to provide clear microscopic images. Additionally, H_2_O_2_ quenches autofluorescence, reducing background noise and false positive signals during imaging.

***Note:*** If the experiment uses transgenic zebrafish lines without pigmentation, such as *casper*, this step can be omitted. It is also possible to depigmentate fish form 1 dpf onward using 0.003% 1-phenyl-2-thiourea (PTU) in 1x E3 and then this step can be omitted. Depigmentation using PTU is not recommended for studies of brain as the chemical actions of PTU on the brain morphology and function are unknown.

1. Collect up to 20 larvae in a 2 mL tube with a flat or round bottom.
2. Just before starting, prepare a clearing solution of 1x KOH + 3% H_2_O_2_ in MilliQ H_2_O.
3. Remove PBS from each tube with fixed larvae and add 1 mL of clearing solution per tube. Incubate at 20°C–22°C until the tissue is cleared (for larvae at 5 dpf, this takes approximately 45 min).

Keep the tubes open during this time.

4. Rinse 2x with PBS and store in PBS at 4°C or proceed immediately to the next step.

### Permeabilization, antigen retrieval, and blocking

**Timing: 6 h + 24 h**

In this step, we describe the first, preparatory steps of the whole-mount immunofluorescence procedure, including tissue permeabilization, washing, antigen retrieval and blocking against nonspecific antibody binding. Triton X-100 and TWEEN 20 are nonionic detergents, enhancing tissue penetration and reducing surface tension respectively, while DMSO removes lipids from cell membranes.

5. Wash the sample tissues (at 20°C–22°C).
  a. Wash cleared samples in PBSTr solution 2x for 1 h.
  b. Wash samples in PBSTr-DMSO solution for 1 h.
  c. Wash samples in PBSTr-DMSO-Tween solution for 1 h.
  d. Wash samples in PBSTr solution 2x for 1 h.
6. Perform antigen retrieval. Troubleshooting 1.
  a. Wash samples with 150 mM Tris.
  b. Add fresh 150 mM Tris and incubate for 5 min at 20°C–22°C.
  c. Incubate for 15 min at 70°C using a thermomixer.
  d. Bring to 20°C–22°C before proceeding with the next step.
7. Block samples against non-specific antibody binding. Troubleshooting 2.
  a. Prepare a blocking solution by diluting the GDB-Tr stock to 1x in PBS.
  b. Incubate samples in blocking solution overnight at 20°C–22°C.

***Note:*** An alternative blocking solution of 0.2% Triton X-100, 20% DMSO, 0.3 M glycine and 6% goat or donkey serum in PBS can also be used, and may work better for some antibodies. Glycine binds aldehydes, and therefore reduces non-specific antibody binding, and can improve the quality of the staining. Serum blocks unspecific binding of the secondary antibody to the tissue, thus, the serum should be used from species that the secondary antibody comes from, e.g. goat serum should be used for blocking when the goat secondary antibodies are used for detection.

### Washing and primary antibody incubation

**Timing: 2 h + 3 days**

This step clears the residue of blocking solution and incubation with the primary antibody. The addition of heparin to the buffer increases antibody diffusion in tissue.

8. Wash samples by incubating them for 2 × 1 h at 20°C–22°C in PBST-heparin solution.
9. Incubate samples with the primary antibody. Troubleshooting 1.
  a. Prepare a 1:200 solution of the rabbit polyclonal anti-pRps6 antibody in PBST-heparin.
    ***Note:*** For staining with alternative antibodies, higher or lower dilutions might be more optimal. Follow the manufacturer’s suggestions for each antibody.
  b. Incubate with antibody solution for 72 h at 20°C–22°C.

### Washing and secondary antibody incubation

**Timing: 15 min + 3 days**

This step clears the residue of primary antibody and incubate with the secondary antibody.

10. Wash samples in PBST-heparin once.
11. Incubate samples in PBST-heparin overnight at 20°C–22°C.
12. Incubate samples with secondary antibody.
  a. Prepare a 1:1000 solution of the Alexa Fluor 488 antibody in PBST-heparin.
    **CRITICAL:** When using the alternative blocking solution containing goat or donkey serum, remember to match the source species of the serum to the host species of the secondary antibody (e.g. when using a goat anti-rabbit secondary antibody, block the samples in goat serum).
  b. Remove the PBST-heparin solution and add the antibody solution.
  c. Incubate with antibody solution for 48 h at 20°C–22°C in darkness (e.g., cover with aluminum foil).
    **CRITICAL:** From this point onwards, keep samples protected from light.

### Washing and mounting

**Timing: 2 days + 15 min**

This step clears the residue of secondary antibody and mounting samples in a glycerol-based medium for long-term storage.

13. Wash samples 2x in PBST-heparin.
14. Incubate samples in PBST-heparin overnight at 20°C–22°C.
15. Incubate samples in a glycerol gradient.
  a. Incubate in 30% glycerol in PBS for at least 2 h at 20°C–22°C (until the larvae fall to the bottom of the tube).
  b. Incubate in 50% glycerol in PBS for at least 2 h at 20°C–22°C (until the larvae fall to the bottom of the tube).
  c. Incubate in 70% glycerol in PBS overnight at 20°C–22°C (until the larvae fall to the bottom of the tube).
16. Transfer samples to mounting medium and store at 4°C in the dark until imaging.

### Imaging with light-sheet microscopy

**Timing: variable (approx. 1 min per sample)**

This step explains the procedure of imaging zebrafish larvae with light-sheet microscopy.

17. Heat up the previously prepared 2% agarose solution to melt it. Keep it in a heat block at 55°C–60°C.
18. Wash samples with PBS to remove the mounting medium.

***Note:*** When imaging the deeper structures of the brain, it might be necessary to remove the eyes and skin in order to increase the light penetration to the region of interest.

19. Place samples in a glass capillary tube filled with agarose.
20. Place the capillary in the imaging chamber filled with PBS.

***Note:*** In this study, samples were imaged using a Zeiss Lightsheet Z.1 microscope (40× water immersion objective, NA = 1.3) at following settings: pivot scan, 1024 × 1024 pixel-resolution, Z-step size 0.5 μm, laser line 488 nm intensity 8%.

***Note:*** Following imaging, samples can be placed back in the mounting medium and stored long-term at 4°C in the dark.

## Expected outcomes

**Figure 2:**
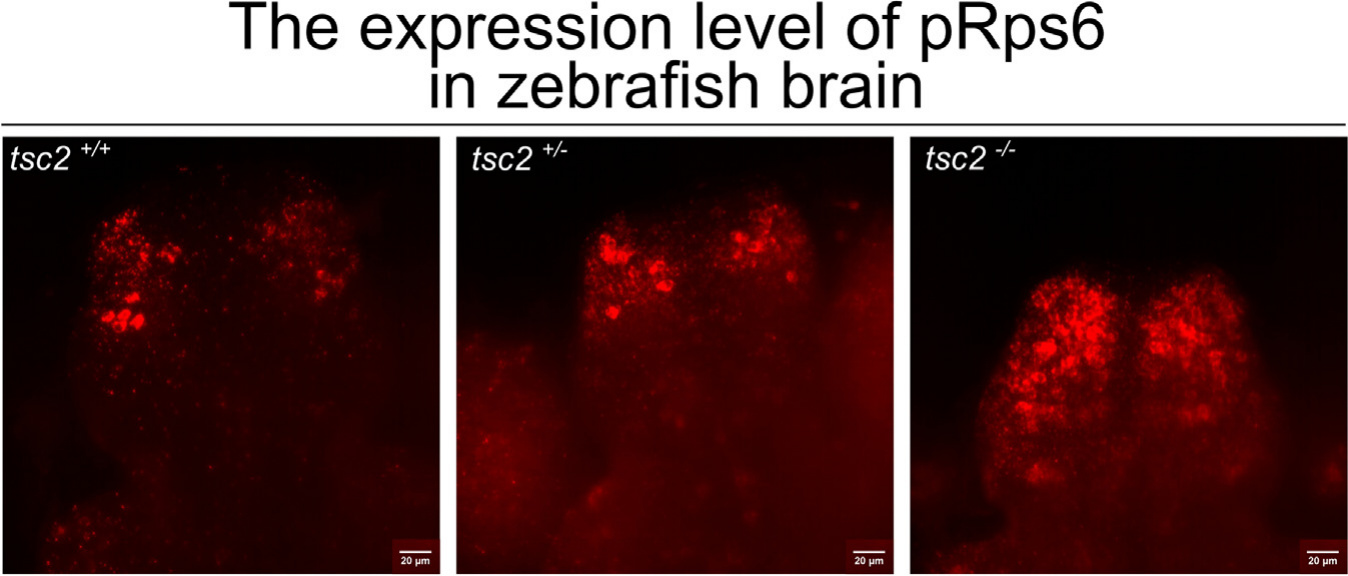
Expression of P-Rps6 in *tsc2-*zebrafish larval brain at 5 dpf Representative images of P-Rps6-positive cells in the pallium, showing differences in expression between genotypes.

This protocol provides methodology for visualization of protein expression patterns in whole zebrafish larval brain. In case of P-Rps6, the fluorescent signal is visible in the cytoplasm (Figure 2).

## Quantification and statistical analysis

**Figure 3:**
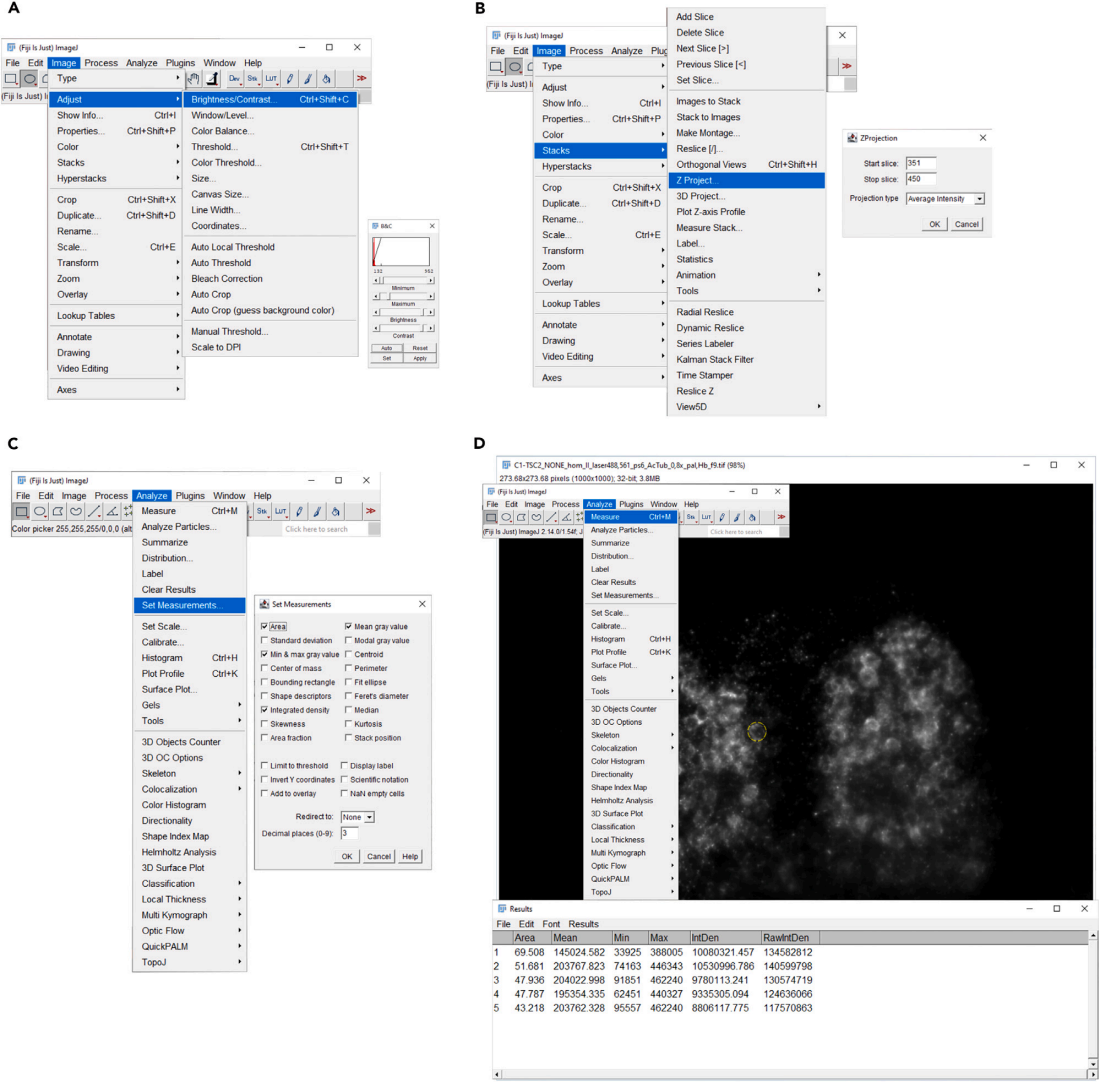
Quantification of P-Rps6 levels in the pallium performed in Fiji software (A) After loading the resized image stack into the Fiji software, in the brightness/contrast level adjustment, set the minimum and maximum value displayed on the image. These values depend on the fluorescence intensity of the images and must be selected individually for the experiment. All images must have the same values set. Therefore, after step B, the images must be verified and checked to make sure that the brightness is not too high and that single positive P-Rps6 cells are visible. (B) This step requires selecting the brain area of interest. Select an equal number of slices from each sample in the stack. Then sum the slices using the projection type: Average Intensity. (C) Set the measurements needed for analysis: Average Intensity, Area, etc. (D) Click the oval function to draw a circle around the positive P-Rps6 cell and measure the parameters. Do this for several representative cells per image. The average value of the measurements will be used to calculate the fluorescence intensity per cell.

Quantification can be performed in Fiji software (Figure 3). Using the Measure tool (set measurements: area, mean fluorescence, min. and max. values), manually select cells in your region of interest, and record the cell size and signal intensity. The number of signal-positive cells can be counted manually. Afterwards, statistical analysis can be performed in RStudio. Check for equality of variance and normality of residuals using Levene’s test and Shapiro-Wilk test, respectively. If those assumptions are met, use an ANOVA test with post-hoc TukeyHSD for data analysis; if they are not, use the Kruskal-Wallis test with *post-hoc* Wilcoxon test to correct for multiple comparisons.

## Limitations

The main limitation of this protocol is the limited availability of antibodies designed for detection of zebrafish proteins. Therefore, the conservation of detected epitope between zebrafish and mammalian orthologs should be assessed and taken into account.

## Troubleshooting

### Problem 1

Lack of or weak fluorescent signal (can be related to step 6 or 9).

### Potential solution

• Test whether the antigen retrieval step is required. For some proteins, the temperature or the buffer in which this step is performed might also need to be adjusted.

OR

• Increase the concentration of primary antibody.

### Problem 2

High background fluorescence (related to step 7).

### Potential solution

• Make sure you used the correct blocking solution; particularly when using a blocking solution containing goat or donkey serum, make sure to match it to the host species of the secondary antibody.

## Resource availability

### Lead contact

Further information and requests for resources and reagents should be directed to and will be fulfilled by the lead contact, Justyna Zmorzynska (j.zmorzynska@imol.institute).

### Technical contact

Technical questions on executing this protocol should be directed to and will be answered by the technical contact, Justyna Zmorzynska (j.zmorzynska@imol.institute).

### Materials availability

This study did not generate new unique reagents. The fish mutant and transgenic lines are protected under material transfer agreement with the institutions that generated the lines. Upon appropriate agreement with these institutions, they can be requested from the lead contact.

### Data and code availability

This study did not generate datasets or codes.

## Acknowledgments

We thank Kevin Ess (Vanderbilt University) for the *tsc2*^*vu242/+*^ zebrafish line, the IIMCB ZCF for assistance with the adult fish, and the IIMCB MCF for sharing the Lightsheet Z.1. This work was supported by an OPUS grant no. 2020/37/B/NZ3/02345 from National Science Centre, Poland. For the purpose of open access, the author has applied a CC-BY public copyright license to any Author Accepted Manuscript (AAM) version arising from this submission.

## Author contributions

This protocol was developed and optimized by J.Z. and O.D. The detailed procedure was written and edited by O.D., T.D., and J.Z. Resources and funding were secured by J.Z.

## Declaration of interests

The authors declare no competing interests.
